# Suggestion of a new standard in measuring the mandible via MRI and an overview of reference values in young women

**DOI:** 10.1007/s10006-023-01153-7

**Published:** 2023-04-26

**Authors:** Leonie Carina Ibald, Veronica Witte, Fank Klawonn, Rupert Conrad, Martin Mücke, Julia Sellin, Marcus Teschke

**Affiliations:** 1 Centre for Rare Diseases Bonn (ZSEB), University Hospital Bonn, Bonn, Germany; 2 Cognitive Neurology, University Medical Center Leipzig, Leipzig, Germany; 3 Max Planck Institute for Cognitive and Brain Sciences, Leipzig, Germany; 4 Biostatistics Research Group, Helmholtz Centre for Infection Research, Braunschweig, Germany; 5 Department of Computer Science, Ostfalia University, Wolfenbüttel, Germany; 6 Department of Psychosomatic Medicine and Psychotherapy, University Hospital Muenster, Muenster, Germany; 7 Institute for Digitalization and General Medicine, University Hospital RWTH Aachen, Aachen, Germany; 8 Centre for Rare Diseases Aachen (ZSEA), University Hospital RWTH Aachen, Aachen, Germany; 9 Dept. of Maxillofacial Surgery, Parkklinik Manhagen, Großhansdorf, Germany

**Keywords:** AICR, MRI, Morphology, Mandible, LIFE study

## Abstract

**Purpose:**

Adult idiopathic condylar resorption (AICR) mainly affects young women, but generally accepted diagnostic standards are lacking. Patients often need temporomandibular joint (TMJ) surgery, and often jaw anatomy is assessed by CT as well as MRI to observe both bone and soft tissue. This study aims to establish reference values for mandible dimensions in women from MRI only and correlate them to, e.g., laboratory parameters and lifestyle, to explore new putative parameters relevant in AICR. MRI-derived reference values could reduce preoperative effort by allowing physicians to rely on only the MRI without additional CT scan.

**Methods:**

We analyzed MRI data from a previous study (LIFE-Adult-Study, Leipzig, Germany) of 158 female participants aged 15–40 years (as AICR typically affects young women). The MR images were segmented, and standardized measuring of the mandibles was established. We correlated morphological features of the mandible with a large variety of other parameters documented in the LIFE-Adult study.

**Results:**

We established new reference values for mandible morphology in MRI, which are consistent with previous CT-based studies. Our results allow assessment of both mandible and soft tissue without radiation exposure. Correlations with BMI, lifestyle, or laboratory parameters could not be observed. Of note, correlation between SNB angle, a parameter often used for AICR assessment, and condylar volume, was also not observed, opening up the question if these parameters behave differently in AICR patients.

**Conclusion:**

These efforts constitute a first step towards establishing MRI as a viable method for condylar resorption assessment.

## Introduction

Adult idiopathic condylar resorption (AICR) is a temporomandibular joint (TMJ) disease also known as cheerleaders’ syndrome or progressive condylar resorption. It mostly affects young women aged 15–35 years [1, 2], and other surveys propose a range of 11–45 years [3]. The term cheerleader’s syndrome refers to the perception that it mostly affects thin young women taking part in sports [4]. Based on a survey by Handelman and Greene, it is estimated that orthodontists see 1 case of idiopathic condylar resorption in 5000 patients [5].

AICR is often associated with a retruded lower jaw, a class II occlusational relationship, and an anterior open bite [4], while it remains unclear if these changes are caused by changes of the condyle or if they are to be perceived as one of many risk factors. Patients might also report progressive changes of their facial features that are accompanied by pain or cracking of the TMJ [4], headaches, malocclusion, or a decreased range of motion of the jaw [1]. According to Wolford and Cardenas, about 25% of patients with AICR experience no symptoms of TMJ disease at all [6].

Some authors use the term AICR in a way that is not clearly defined and include osteoarthritic changes of the TMJ into their definition [7, 8]. Contrary to that, some authors particularly exclude cases with osteoarthritis, previous craniomaxillofacial surgery, or trauma of the TMJ, to define AICR as truly idiopathic [4, 9, 10]. While some authors want to exclude patients that have undergone orthognathic surgery from the diagnosis, others propose that orthognathic surgery might be a triggering event in the development of AICR and therefore might be part of a pathomechanism leading to the disease. One might also argue that currently AICR is likely under-diagnosed and patients have to undergo a long journey to receive their diagnosis, on which journey they may have received orthognathic surgery anyway, so excluding subjects that have had orthognathic surgery could distort scientific results. On top of that, Wolford supposes that the relationship between orthognathic surgery and AICR might only be coincidental but not causally determined in any direction [4], which further highlights the complexity of the topic and how little we know to this date. It also emphasizes inconsistencies in the definition of AICR, which complicate scientific research and clinical diagnosis, and which are based on gaps in our knowledge regarding AICR. Sansare et al. mention in their systematic review that in research, cases of AICR should be well defined and not mixed up with other diseases [11]. However, this is problematic since the diagnostic process is further complicated as a result of a lack of known laboratory tests that are specific for AICR [4].

As Sansare et al. proposed in 2015, further investigation on the presentation of the TMJ in 3D imaging procedures is needed [11]. It is particularly important to note that the progression of the disease, estimated to be 1.5 mm/year, might be underestimated by only looking at 2D images of the mandible [11]. Therefore, 3D imaging, e.g., via MRI or CT scan, is an important improvement to assess the development and progression of AICR, and condylar resorption in general.

As the variety of radiographic findings in patients with AICR is substantial in the literature and includes both bones and soft tissues [11], we consider MRI to have great potential to assess the disease, as both bones and soft tissues can be observed, and the 3D morphology of the condyle can be visualized.

We used datasets of the LIFE-Adult-Study [12], a cohort study initiated by the University of Leipzig, which include MR images of the head suitable for the segmentation of 3D models, and a wide variety of background information to assess possible correlations to the morphology of the mandible.

## Aims

The primary aim of this study was to establish reference values for measurements in segmented models of an MRI of the mandible in young women aged 15–40, i.e., the population mostly affected by AICR [1, 2], in order to gain a base line for further research, and to establish MRI measurements as an efficient tool for mandible assessment. As the definition of AICR is inconsistent throughout research and the pathogenesis of AICR is only poorly understood, we chose to include data of a large healthy cohort and put a special focus during evaluation on participants whose mandibles show measurements consistent with AICR (i.e., a low condylar volume or a small SNB angle). We assessed SNB angle, condylar volume, the volume of the processus muscularis, ramus length, depth of the antegonial notch, and length and width of the condyle. To the authors’ knowledge, there are no studies available yet that evaluate these measurements of the mandible as presented in MRI.

The second aim was to compare MRI measurements to other parameters surveyed by LIFE, such as blood count, vitamin-D-levels, dietary habits, or anthropometric data, to evaluate if there are any connections to be made between the morphology of the mandible and another parameter, thus to gain new hints on the origins of condylar resorption, and as a baseline for laboratory tests that might be interesting candidates to help the diagnostic process.

## Methods

### Data set

The age bracket of AICR-affected women is often given as 15–35 years, but 11–45 years are also reported in some studies [3]. We therefore compromised and defined the inclusion criteria as women aged 15–40 years. The data of 171 participants from the collective of the LIFE-Adult study [12] that had an MRI of the head were included in this study. Thirteen participants were subsequently excluded because of incomplete data, or an MRI of insufficient quality or incomplete presentation of the mandible, resulting in a final data set of 158 participants, aged 19–40 years. The parameters that were requested from the LIFE-Adult study database for exploration in this study are listed in Table 1.

**Table 1:** Assessments requested of LIFE database. Theses parameters were requested of the LIFE database and included into our analysis to assess possible further parameters for the diagnosis of AICR

Age and gender of the participants
Medical anamnesis, medication history, family anamnesis
Alcohol consumption, tobacco consumption, tobacco consumption, and passive smoking
Anthropometry, body-mass-index, visceral and subcutaneous adipose tissue (VAT; SCAT)
Diabetes
Three-Factor Eating Questionnaire—eating behavior, Food Frequency Questionnaire, Yale Food Scale
International Physical Activity Questionnaire
Pittsburgh Sleep Quality Index, Epworth Sleepiness Scale
Oral health
Complete blood count, HbA1c, adiponectin, leptin, bioavailable leptin, fatty acid-binding protein (FABP), ghrelin, thyroid-stimulating hormone (TSH), alkaline phosphatase, phosphate, calcium, osteocalcin, beta-CTx, parathormone, 25-OH-vitamine D3, luteinizing hormone (LH), testosterone, testosterone (LC–MS/MS), follicle-stimulating hormone (FSH), estradiol, estradiol (LC–MS/MS), progesterone (LC–MS/MS), sex hormone-binding globulin (SHBG), dehydroepiandrosteronesulfate (DHEAS-S), dehydroepiandrosteronesulfate (DHEAS-S) (LC–MS/MS), inhibin-B, anti-Mullerian hormone, high-sensitive C-reactive protein (hs-CRP), interleukin-6

### MRI segmentation

MRI results were available in the NIFTI (Neuroimaging Informatics Technology Initiative) file format, as they were originally intended to be used for neuroimaging [12]. The sequence used was MPRAGE (Magnetization Prepared Rapid Gradient Echo).

We used the software Mimics InPrint 3.0 and created models of the mandibulae of the participants by a process that was in parts automatized, and refined and corrected manually by looking into every slice. A similar way of segmentation was used by Cevidanes et al. [13].

First, the contrast of the display was adjusted to show the outlines of the bones most clearly. Then, a thresholding tool was used to include the corticalis of the bone as accurately as possible. After that, the segmented model was corrected manually in all three planes by going through every slice. As a last step, the model was smoothed with the option “low” to minimize step-like formations.

### Measurements

**Fig. 1:**
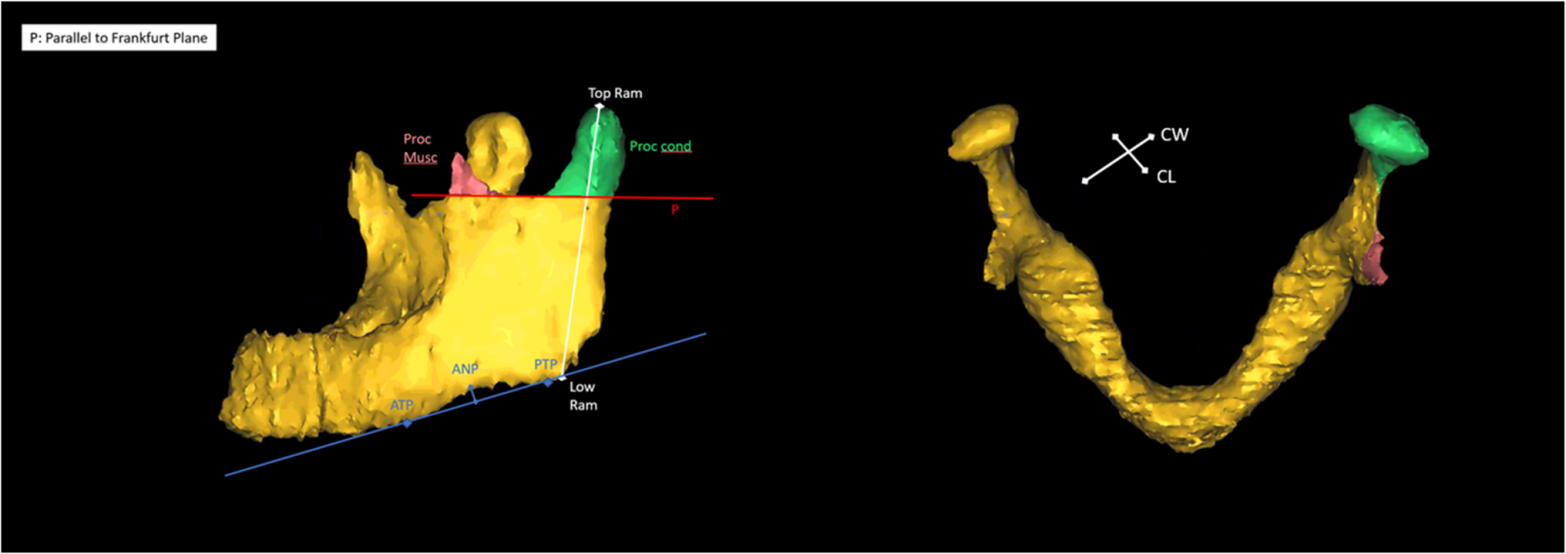
Illustration of the measuring points. An example of a 3D model of the mandible. The red line (P) represents a plane parallel to Frankfurt plane, touching the most inferior point of the mandibular notch, that was used to set the inferior boundary of the muscular processus (Proc. Musc.), in red, and the condylar processus (Proc. Cond.) in green. Also the measurements between the most superior (Top Ram) and inferior points (Low Ram) of the mandibular ramus are presented here in white. The blue line shows the line between the anterior and posterior tangent point (ANP and PNP) that was used to assess the depth of the antegonial notch (ANP) in a 90° angle to the blue line. Also the condylar width (CW) and condylar length (CL) are illustrated

As the major radiographic findings in AICR include changes of the SNB angle, decreased height of ramus and condyle and a reduced condylar volume [11], we chose to measure those features in our study. An overview of the features measured with the corresponding measuring points is given in Table 2 and illustrated in Fig. 1.

**Table 2:** Measurements applied to our 3D models of the mandibles. This table gives an overview of all morphological measurements of the mandible assessed in our study, the abbreviations used and the exact definitions of the measured parameters

Term	Abbreviation	Location
Sella	S	Middle of the sella turcica
Nasion	N	Vertex of the concave of the nasal root as seen in the lateral profile
Gonion	B	Vertex of the concave mental part of the mandible as seen in the lateral profile in the centre of the alveolar processus
Top ramus	Top Ram	Bilateral; Most cranial point of the condylar processus
Low ramus	Low Ram	Bilateral; Vertex of the jaw angle
Antegonial notch	AN	Bilateral; Most cranial point of the antegonial notch
Anterior tangent point	ATP	Bilateral; Anterior point of a caudal tangent on the alveolar processus
Posterior tangent point	PTP	Bilateral; Posterior point of a caudal tangent on the alveolar processus
SNB angle	SNB	Angle between S, N, and B
Ramus length		Bilateral; Distance between Top Ram and Low Ram
Depth of the antegonial notch	AND	Bilateral; Distance between AN and the tangent on ATP and PTP in a right angle
Condylar width	CW	Bilateral; Condylar width latero-medial direction in axial view
Condylar length (antero-posterior direction)	CL	Bilateral; Condylar length (antero-posterior direction) in axial view

The volumes of the condylar and muscular processes were assessed with the tool “osteotomy”. For that, a cutting plane parallel to the Frankfurt plane was placed on the most caudal point of the incisura mandibulae.

The measurements were taken after the segmented model was adjusted in Frankfurt plane horizontally. Frankfurt plane is determined by the cranial edges of both the meatus acustici interni and the caudal edges of the orbital bone.

The mandibulae were classified as presented in Table 3, based on the general approach used in clinical practice. An angle class I SNB angle is considered normal and lies between 78 and 82°. An SNB angle under 78° is angle class II and considered small or retrognathic. Angle class III SNB angles are over 82° and considered large or prognathic.

**Table 3:** Classification based on SNB angle. This table shows the classification of the mandible based on the SNB angle and explains whether mandibles falling into these categories are considered retrognathic, prognathic, or normal

Class	Classification of the mandible	SNB angle
I	Normal	78–82°
II	Retrognathic/ “small”	≤ 78°
III	Prognathic/ “large”	≥ 82°

### Software used

Mimics InPrint 3.0 was used for the process of segmentation. The software ProPlan 3.0.1 was used for measuring the segmented 3D models of the mandibles.

### Statistical methods

Numbers were rounded to two decimal places unless stated otherwise. For testing associations of numerical variables with binary variables, the *t*-test and the Wilcoxon-Mann–Whitney test were applied. In case of non-numerical variables including the discretization into the categories small, average and large the ${\chi }^{2}$-test was used. None of these *p*-values was reported because they were never below 0.05. The *p*-values in Table 4 were computed with the binomial test, assuming that the reference range reflects 95% of the healthy population. Correlations are stated as Pearson correlation coefficients. Kendall correlations were also computed to account for possible outliers but no relevant differences to Pearson correlations could be observed. Weight normalized condylar volumes were computed as condylar volume divided by the weight of the person.

**Table 4:** Number of participants with elevated platelet counts. “Small” refers to an SNB ≤ 78° or a condylar volume smaller than the first quartile. “Average” refers to an SNB angle between 78 and 82° or a condylar volume between the first and third quartile. “Large” refers to an SNB ≥ 82° or a condylar volume over the third quartile. *p*-values for the null hypothesis that the corresponding figures occur in a healthy sample are given in brackets

	Small	Average	Large
SNB	5 (0.019)	4 (0.076)	1 (0.555)
Condylar volume left	5 (0.003)	4 (0.131)	1 (0.637)
Condylar volume right	7 (0.0005)	2 (0.584)	1 (0.637)
Condylar volume left weight normalized	4 (0.018)	5 (0.046)	1 (0.637)
Condylar volume right weight normalized	7 (0.0005)	3 (0.310)	0 (1)

## Results

Changes in SNB angle and condylar volume as well as changes in height of the mandibular ramus are parameters that are associated with condylar resorption [11]. Therefore, we included these measurements into our study. As the length and width of the condyle (2D parameters in the axial view) are linked to the condylar volume and can be assessed without segmenting a 3D model of the mandible, and might therefore be helpful in a clinical routine setting, we included these parameters as well.

Of 158 participants, 33 had a class II SNB angle of ≤ 78°, 59 had a class I SNB angle of 78–82°, and 67 had a class III SNB angle of ≥ 82°. The minimal SNB was 59.9°, the maximum 89.1°.

We defined a normal condylar volume as between the first and third quartile; according to this, a “small” condylar volume was below the first quartile (1311.25 mm^3^ on the right, 1387.47 mm^3^ on the left), and a “large” volume above the third quartile (1855.37 mm^3^ on the right, 1873.65 mm^3^ on the left). The minimal volume measured on the right side was at 656.99 mm^3^, on the left side at 769.48 mm^3^. The maximal volume measured was 3234.58 mm^3^ on the right side and 2957.35 mm^3^ on the left. Our measurements of the condylar volume are shown in Table 5.

**Table 5:** Condylar volumes. The volumes of the right and left condyle and distribution parameters are presented in this table

	Condylar volume right	Condylar volume left
Minimum	656.99 mm^3^	769.48 mm^3^
First quartile	1311.25 mm^3^	1387.47 mm^3^
Third quartile	1855.37 mm^3^	1873.65 mm^3^
Maximum	3234.58 mm^3^	2957.35 mm^3^
Average	1584.21 mm^3^	1641.67 mm^3^
SD	415.77	389.30

Condylar volumes of both sides (verage right condylar volume: 1584.21mm³, SD 415.77, average left condylar volume: 1641.67 mm³, SD 389.30) and the SNB angle (average 81.1° SD 4.05) were nearly normally distributed.

**Fig. 2:**
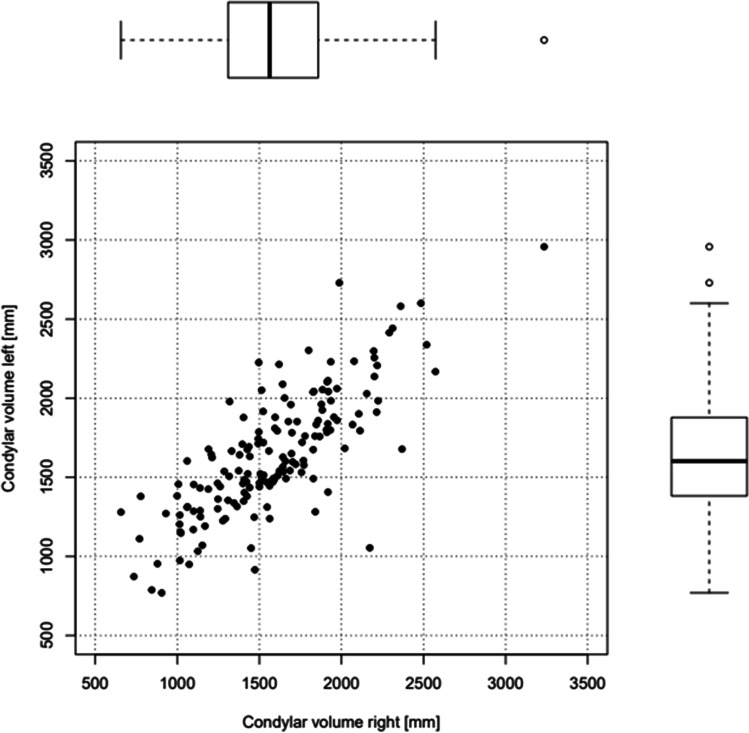
Correlation of condylar volumes. This represents the correlation and distribution of left and right condylar volume

Condylar volume on the left and right side showed a correlation of 0.77 (Fig. 2), which is surprisingly low given the bilateral symmetry of the mandible.

The average length of the right condyle was 9.08 mm (median 8.85 mm; SD 1.54 mm) with a minimum of 5.7 mm and a maximum of 19.5 mm. On the left condyle, the average length was 9.11 mm (median 8.9 mm; SD 1.30 mm) with a minimum of 5.9 mm and a maximum of 13.5 mm. For both sides, the average length was 9.09 mm with a median of 8.9 mm and a standard deviation of 1.42 mm.

The average width of the right condyle was 17.74 mm (median 17.85 mm; SD 2.16 mm) with a minimum of 12.2 mm and a maximum of 22.7 mm. On the left, the average width of the condyle was 18.03 mm (median 18.1 mm; SD 1.99 mm) with a minimum of 12.4 mm and a maximum of 23.1 mm. The average for both sides combined was 17.88 mm with a median of 18 mm and a standard deviation of 2.08 mm.

We assessed an average mandibular ramus length on the right side of 53.54 mm (median 53.5 mm; SD 4.19 mm) with a minimum of 38.1 mm and a maximum of 63.7 mm. On the left, the mean ramus length was 54.22 mm (median 54.2 mm; SD 4.17 mm) with a minimum of 42.1 mm and a maximum of 65.8 mm. On both sides taken together, the mean ramus length was 53.88 mm with a median of 53.85 mm and a standard deviation of 4.19 mm.

To our knowledge, there are no referential values for antegonial notching and the volume of the muscular processus, neither for CT imaging nor for MRI. We therefore attempted to extract these from the dataset. We measured a mean volume of the muscular processus on the right side of 210.21 mm^3^ (median 179.57 mm^3^; SD 139.34 mm^3^) with a minimum of 10.73 mm^3^ and a maximum of 645.68 mm^3^. On the left, the average volume was 212.44 mm^3^ (median 188.47 mm^3^; SD 138.70 mm^3^) with a minimum of 6.27 mm^3^ and a maximum of 949.46 mm^3^. For both sides, the average volume of the muscular processus was 211.32 mm^3^ (median 188.47 mm^3^; SD 138.80 mm^3^). However, the segmentation of the muscular processus was limited by the presentation in MRI, as the structure of the processus is very thin and likely not to be captured in the slices properly. On the right side 3 and on the left side 4 processi musculari could not be segmented. Also, the manual correction of the outline during segmentation was hampered as the differentiation between bone and the soft tissue around it was vague. Therefore, we expect our measurements of the muscular processus to be more of an estimate.

The average depth of the right antegonial notch was 2.02 mm (median 2.15 mm; SD 0.99 mm) with a minimum of 0 mm and a maximum of 5.7 mm. On the left, the average was 1.68 mm (median 1.8; SD 0.98 mm) with a minimum of 0 mm and a maximum of 6 mm. The average of both sides taken together was 1.85 mm with a median of 2 mm and a standard deviation of 0.99 mm.

To the authors’ knowledge, none of these parameters has been assessed in MR imaging of the healthy jaw to this date. Ramus length, condylar length and width, and condylar volume have been assessed previously, but only in CT scans [14–17], so the measurements given above represent new referential values for measurements of the mandible in MRI. For a summary and overview of all resulting measurements, see Table 6.

**Table 6:** Measurements of the mandible in MRI. This gives an overview on all morphological parameters assessed and their statistical distribution parameters. All parameters measured are given in mm

Parameter	Mean/average	Median	Minimum	Maximum	Standard deviation
SNB [degrees]	81.1	81.4	59.9	98.1	± 4.05
Volume condylar processus [mm^3^]	1612.94	1582.78	656.99	3234.58	± 403.14
Volume condylar processus right [mm^3^]	1584.21	1560.45	656.99	3234.58	± 415.77
Volume condylar processus left [mm^3^]	1641.67	1599.79	769.48	2957.35	± 389.30
Volume muscular processus [mm^3^]	211.32	188.47	6.27	949.46	± 138.80
Volume muscular processus right [mm^3^]	210.21	179.57	10.73	645.68	± 139.34
Volume muscular processus left [mm^3^]	212.44	188.47	6.27	949.46	± 138.70
Condylar length [mm]	9.09	8.9	5.7	19.5	± 1.42
Condylar length right [mm]	9.08	8.85	5.7	19.5	± 1.54
Condylar length left [mm]	9.11	8.9	5.9	13.5	± 1.30
Condylar width [mm]	17.88	18	12.2	23.1	± 2.08
Condylar width right [mm]	17.74	17.85	12.2	22.7	± 2.16
Condylar width left [mm]	18.03	18.1	12.4	23.1	± 1.99
Antegonial notch [mm]	1.85	2	0	6	± 0.99
Antegonial notch right [mm]	2.02	2.15	0	5.7	± 0.99
Antegonial notch left [mm]	1.68	1.8	0	6	± 0.98
Ramus length [mm]	53.88	53.85	38.1	65.8	± 4.19
Ramus length right [mm]	53.54	53.5	38.1	63.7	± 4.19
Ramus length left [mm]	54.22	54.2	42.1	65.8	± 4.17

We next analyzed if SNB angle and condylar volume are correlated, as both small SNB angles as well as low condylar volume are used to assess the presence of AICR. Of note, there was no significant correlation between SNB angle and condylar volumes. The Pearson correlation between the left condylar volume and SNB was 0.075, while the correlation between the right condylar volume and SNB was 0.153. For comparison, the Pearson correlation between the condylar volumes of both sides of the mandible was 0.78 (Fig. 2).

To take into account other general physiological parameters that might influence jaw physiognomy, we assessed also correlations between SNB angle or condylar volume and height or weight of the participants as presented in Table 7. The SNB angle correlated with a factor of 0.19 to the height and 0.14 to the weight, so there is a slightly stronger correlation to height. The condylar volumes on the right side correlated with a factor of 0.18 with the height and a slightly higher factor of 0.22 with the weight. On the left, the correlation with height was 0.19 and with weight was 0.24, so condylar volumes seem to correlate slightly more with the weight of the participants.

**Table 7:** SNB and condylar volume related to height and weight. This table shows the correlation factors of the condylar volumes on both sides and SNB to the height and weight of the participants

	Height	Weight
SNB	0.19	0.14
Condylar volume right	0.18	0.22
Condylar volume left	0.19	0.24

Nevertheless, the overall correlations were only weak, so the height and weight of the participants do not seem to be the defining parameters for condylar volume or SNB angle.

We were not able to find any relevant correlations between the SNB angle or condylar volumes and other parameters, like eating habits, physical activity, or various laboratory parameters, likely because our sample of participants was too small to detect more subtle effects (for a list of all parameters analyzed, see Table 1).

We hypothesized that there could be a correlation between sex hormones and small condylar volumes or SNB, as AICR often occurs in young women, suggesting that changes in the hormonal balance might play a role in pathogenesis. Unfortunately, the laboratory parameters for sex hormones were not documented in relation to anamnesis of the menstrual cycle, so we chose not to use this data, as this does not permit the reconstruction of the cycle phase and therefore the interpretation of the values measured is prohibited.

We also took a closer look at parameters indicating bone metabolism like calcium, parathormone, 25-OH-vitamin D, and alkaline phosphatase, as condylar resorption affects the bone and might be accompanied by altered bone metabolism. We looked at markers of inflammation as well, e.g., hs-CRP or IL-6, as these might hint at inflammatory causes of condylar resorption. While no correlations could be observed, it should be mentioned that such a correlation in a pathological situation cannot be ruled out as the data set comprises participants from the general population.

Upon closer inspection of the three groups of small, average, and large SNBs or condylar volumes, we made an interesting observation: Three of our participants reported to be diagnosed with tinnitus in their medical anamnesis, and all three had a low condylar volume under 1300 mm^3^ on both sides. Of note, Alsabban et al. mention the occurrence of tinnitus in association with temporomandibular disorder [1]. Further research to validate this observation might be warranted.

Of the 10 participants that had elevated platelets, most seemed to have a rather small condylar volume and/or SNB angle. A total of 4 out of 10 had a small condylar volume on both sides and 3 out of those 4 had a small SNB angle also.

## Discussion

### Use of MRI of the mandible in clinical practice and research

Nowadays mostly CT scans, panoramic radiographs, and MRI are used especially in preoperative assessment of AICR [1]. MRI is a technique that can provide 3D datasets presenting the soft tissue as well as the bony structures, even though usually CT scans are estimated to be most suitable to show the outlines of the bone. However, in a study by Neubert et al., CT and 3D MRI were established as comparable. Models segmented from MRI tended to be smaller than CT models, but the models all differed less than a millimeter between CT and MRI [18]. Other authors have used MRI to classify TMJ morphology (e.g., as flat, biconvex) [19]. A study by Koehne et al. in 2018 used MRI scans of patients with mucopolysaccharidosis to assess their bone morphology [20]. The characterization of bony structures in MRI is a way to reduce ionizing beams for the patient, and reduce time and cost for medical services, because the CT scans could be spared. Also, MRI is more common to be used in the acquisition of data for research purposes, because there are no ionizing beams and the potential harm for participants is thus reduced. Therefore, we chose to use MRI datasets to assess the measurements of the mandible. To the knowledge of the authors, we are also the first to establish reference values for the measurements of the mandible in MRI.

In this study, we were able to establish a first set of referential values for the measurements of the mandible in MRI in young women. This data can be used not only in further research but also in medical practice to assess the size of the mandible of patients that may have AICR or other forms of condylar resorption, and to estimate whether the patient has rather big or small condyles. This could over time lead to a clearer understanding of which parameters are best suited to predict the presence of AICR.

As we could find no relevant correlation between the SNB angle and condylar volumes, this poses the question if the use of SNB to assess condylar resorption, as defined as the loss of condylar volume, is adequate. However, as mentioned above, correlations could be different in patients suffering from AICR, as the sample population for our study was recruited from the general population. Yang and Hwang measured an average of 71.19° (SD ± 3.17) for the SNB angle in patients with condylar resorption [7], and Troulis et al. measured a mean SNB of 70.1° preoperatively [10] in patients with condylar resorption, which is far below the average of 78–82° and indicates a link between SNB and condylar resorption. However, 32 of the healthy participants in our study have very small SNB angles with the minimum measured being about 60°, shedding doubt on the suitability of SNB angle alone and especially without multiple measurements over a longer period of time to diagnose AICR. A longitudinal study could explore how SNB angles change over time during disease progression in AICR. Further research on this subject seems highly necessary, especially studies including participants already diagnosed with AICR. If SNB further proves insufficient in describing condylar resorption, it enhances the need for 3D imaging of the mandible to assess condylar volumes to diagnose condylar resorption.

### Accuracy of our method

Farronato et al. used a similar technique in their study to assess the condylar volume in 3D models based on cone-beam-CT-scans, where they also used Frankfurt plane as a reference and used the last slice showing the sigmoid notch as limiting plane and cut off the 3D model there. In their healthy control group consisting of 25 participants, the mean volume was 1386.47 mm^3^ (SD ± 455.21 mm^3^) [14], whereas our mean volume was at 1612.94 mm^3^ (SD ± 403.14). Serindere et al. used a similar method and published data for male and female participants, including 66 female participants. The condylar volumes measured by Serindere et al. were 1546.94 mm^3^ (SD ± 286.71) on the right side, and 1526.04 mm^3^ (SD ± 282.55) on the left side [16], and we assessed similar volumes of 1584.21 mm^3^ (SD ± 415.77) on the right side and 1641.67 mm^3^ (SD ± 389.30) on the left side. Lentzen et al. assessed a mean condylar volume of 1353 mm^3^ (SD ± 0.466) on the left and 1291 mm^3^ (SD ± 0.449) on the right [21], which is smaller than our results and the results published by Serindere et al., but is similar to the results of Farronato et al. [14]. Finally, the mean condylar volume was assessed as 1339.65 mm^3^ (SD ± 494.93) from a study with 100 healthy female participants by Al-koshab et al. [17].

Santander et al. assessed mean condylar depths of 8.5–7.5 mm depending on the skeletal class and mean condylar widths of 19.2–20.2 mm [15], which resembles our measurements of a mean condylar length of 9.09 mm (SD ± 1.43) and a mean condylar width of 17.88 mm (SD ± 2.08). In the study by Serindere et al., participants presented a mean condylar length of 7.71 mm (SD ± 1.14) on the right side and 7.57 mm (SD ± 1.15) on the left side [16], which is slightly smaller than the averages of our measurements of the condylar width. They also assessed a mean condylar width on both sides of 16.08 mm (SD ± 2.51 on the right side, SD ± 2.39 on the left side).

Koshab et al. assessed a mean condylar length of 7.11 mm (SD ± 1.03) and a mean condylar width of 17.04 mm (SD ± 2.35) [17].

The ramus length in the healthy control in the study by Farronato et al. was 54.43 mm (SD ± 4.37). We measured a mean ramus length of 53.88 mm (SD ± 4.19).

Taken together, the abovementioned five studies using CT scans observed similar values for condylar volumes, condylar length and width, and ramus length. The similarities between their results and our measurements confirm the accuracy of our method using MRI. Furthermore, a literature review published by de Melo et al. is also consistent with our findings [22]: They published an average medio-lateral dimension of the condyle of 17.04–20 mm, while we found an average condylar width of 17.88 mm. The average antero-posterior dimension was between 5.12 and 9.6 mm, whereas we found a condylar length of 9.09 mm.

In addition, a study by Coombs et al. confirms the validity of MRI-based measurement of the mandible, as they compared MRI, CBCT, and physical measurements of the mandible and found only minor differences between the CBCT-based data and the data acquired by MRI segmentation, while physical measurements were overall larger than both of the other [23].

Marghalani et al. used a different technique to determine the condylar processus, but they measured a slightly larger left condylar volume as well as we did [24], whereas Serindere et al. in [16] measured a larger mean volume of the condylar processus of the right side.

To the authors’ knowledge, there are no studies to this date describing the volumes of the muscular processus and the antegonial notch in healthy participants, by using neither CT scans nor MRI, which makes this the first description of these assessments, even though, as mentioned above, the measuring of the muscular processus proved to be complicated and is likely inaccurate. Further research to put our measurements into context is needed.

### Correlations between morphology of the mandible and other parameters

A surprising finding of our study was that height and weight of the participants correlate only weakly with mandibular size. Furthermore, we found only a very weak correlation between the condylar volumes and SNB angles in general, whereby the correlation with the right condylar volume was almost twice as strong as with the left condylar volume. This is an important observation since it challenges the common assumption that SNB angle is a suitable parameter to assess condylar resorption and warrants further investigation. These results of our study are in contrast to Saccucci et al., who found a significantly larger condylar volume in class III mandibles, compared to classes I and II, and a significantly smaller condylar volume in class II mandibles as in classes I and III [25]. Of note, it should be taken into account that Saccucci et al. used the ANB angle to classify the mandibles, which poses the question of how SNB and ANB angle differ in healthy individuals and in patients suffering from TMJ disease.

We chose to use data from a large cohort study because AICR is so poorly understood in its pathogenesis and often not easily distinguished from other diseases of the TMJ. We hoped to gain hints on the origins of condylar resorption by examining participants with a rather small condylar volume. We tried to use the volume of the condyle and the SNB angle as surrogate parameters and chose a population of participants in that AICR generally occurs and tried to find any hints in the laboratory values, eating habits, or assessments used that might lay ground for further research on the origins of this disease and the underlying pathomechanisms. This aim was hindered by the still too small number of participants, so we could only assess weak correlations between condylar volume and any other parameter. Furthermore, it remains unclear if there are general differences in correlations between parameters between healthy participants and people suffering from AICR. In conclusion, further research on the subject is needed to put our findings into context.

Lastly, we want to point out that we found hints that patients with elevated platelets seem to more often have a smaller condylar volume and retrognathic occlusion/SNB. While this is only a hint we gained from our data, it might nevertheless be worth pursuing further, as a higher count of platelets could indicate an altered coagulation system, and therefore point to poor perfusion of some parts of the body. This could be interesting as it is imaginable that coagulation disorders play a role in condylar resorption.

### Limitations

The first aim of this study was to establish reference values for the measurements of the mandibula in MRI-segmented models. While we obtained reference values for women aged 19–40, this data collection should be expanded to other age groups and all genders to gain an impression of the changes in morphology in different ages and the differences between male and female participants. In this context, hormonal status should also be considered in female participants in the future.

The second aim of this study was to correlate the measurements assessed to other parameters. This was mostly limited by the small number of participants with small SNB and small condylar volume, as participants for the LIFE study were recruited from the general population, and AICR is a relatively rare disease. While we were not able to find correlations between SNB or condylar volume and other parameters, this could be different in people suffering from AICR. In the future, larger studies or studies that specifically recruit participants with diagnosed AICR are needed to search further for indicators of the pathogenesis of the disease.

Also, as our study is based on a previously collected data set, our study lacks a longitudinal approach, which would be essential in the diagnosis of condylar resorption as it is a progressive disease and often only can be diagnosed over the course of time. It would be interesting to see if any parameters or correlations change in connection to changes of the mandibular bone during AICR progression.

Also, minor deviations of the measurements of the condylar volume and SNB might have occurred in our study due to the possibility of probands not occluding their jaw fully during the acquisition of the MR image, as the mandible is mobile in relation to the rest of the skull. These minor inaccuracies are common in studies on this subject; e.g., Farronato et al. used a very similar technique to determine condylar volumes in their study, based on the findings of Schlueter et al. [14, 26]. By using a similar way of determining our cutting plane to assess the condylar volume, the comparison between the accuracy of measurements in MRI and CT scans was improved. Nevertheless, we deem it necessary to employ further studies using methods to eliminate this source of measurement in certainties, e.g., by using a cutting plane only determined by clearly defined anatomic structures of the mandible itself.

## Conclusion

Our results give an overview on the measurements of the mandible of women aged 19–40 years in MR imaging. As MRI is part of standard diagnostics in any TMJ disease to examine soft tissues like the articular discus, while CT scans are additionally used to assess the morphology of the bone, the possibility to assess the bony structures of the TMJ in the MRI as well enables a more effective use of resources, sparing the CT scan, and imposes less radiation on the patient. These considerations make our referential data on MRI-segmented mandibles an important prerequisite to improve patient care while also sparing resources.

Unfortunately, we did not detect any correlations of SNB and condylar volume to laboratory parameters or conspicuous issues regarding lifestyle, medical anamnesis, or oral health, which could be contributed to the relatively small sample size and the fact that participants were recruited from the general population, with only very few, if any, participants likely suffering from AICR.

We did not find a significant correlation between SNB angle and condylar volume, which is particularly interesting, as the SNB angle is often used as a surrogate parameter to assess condylar resorption in clinical practice. It might therefore be warranted to repeat our measurements with people affected by AICR to elucidate if SNB angles in the presence of AICR indeed correlate with the loss of condylar volume, which is the defining element of condylar resorption.

When correlating height and weight of the participants to SNB and the condylar volumes, we only found weak correlations, while SNB correlated more strongly with height and the condylar volumes showed stronger correlations with the weight of the participants, even though the differences are minor.

As we reported above, three of our participants mentioned having a tinnitus and all three of them had low condylar volume on both sides of the mandible. As tinnitus might be associated with temporomandibular disorder [1], we thought this is an interesting observation that could be followed up in future studies.

Ten participants showed elevated platelets in the complete blood count, and we noted that of those 10 participants, 5 had a small SNB angle and tended to have rather small condylar volumes, as can be seen in Table 7. As an elevated number of platelets is associated with a higher risk of a thrombus, it might be conceivable that inhibition of the blood flow in the condylar region caused by elevated platelets could lead to reduced bone metabolism and smaller condylar volumes. A possible connection to AICR could be explored in future studies.

## Data Availability

The datasets generated during and/or analyzed during the current study are available from the corresponding author on reasonable request.
