# Correction to: Suggestion of a new standard in measuring the mandible via MRI and an overview of reference values in young women

**DOI:** 10.1007/s10006-023-01164-4

**Published:** 2023-06-10

**Authors:** Leonie Carina Ibald, Veronica Witte, Fank Klawonn, Rupert Conrad, Martin Mücke, Julia Sellin, Marcus Teschke

**Affiliations:** 1 Centre for Rare Diseases Bonn (ZSEB), University Hospital Bonn, Bonn, Germany; 2 Cognitive Neurology, University Medical Center Leipzig, Leipzig, Germany; 3 Max Planck Institute for Cognitive and Brain Sciences, Leipzig, Germany; 4 Biostatistics Research Group, Helmholtz Centre for Infection Research, Braunschweig, Germany; 5 Department of Computer Science, Ostfalia University, Wolfenbüttel, Germany; 6 Department of Psychosomatic Medicine and Psychotherapy, University Hospital Muenster, Muenster, Germany; 7 Institute for Digitalization and General Medicine, University Hospital RWTH Aachen, Aachen, Germany; 8 Centre for Rare Diseases Aachen (ZSEA), University Hospital RWTH Aachen, Aachen, Germany; 9 Dept. of Maxillofacial Surgery, Parkklinik Manhagen, Großhansdorf, Germany

**Correction to: Oral and Maxillofacial Surgery (2023)**

10.1007/s10006-023-01153-7

The original article contains an error. Table 5 has been modified due to the mix up of numbers during the proofing process.

The original article has been corrected.

